# Supplementary material for: A Critical View of the Application of the APEX Software (Aqueous Photochemistry of Environmentally-Occurring Xenobiotics) to Predict Photoreaction Kinetics in Surface Freshwaters
Source: Molecules. 2019 Dec 18;25(1):9. doi: 10.3390/molecules25010009 (PMC7017383; doi:10.3390/molecules25010009)
Supplement: Supplementary file 1 [file molecules-25-00009-s001.zip › 3.2.4_gcc-4.4.0.zip!/3.2.4_gcc-4.4.0/3.2.4_gcc-4.4.0/doc/octave/html/interpreter/Running-Octave.html]

Running Octave - Untitled


Next: Simple Examples,
Up: Introduction


---

### 1.1 Running Octave

On most systems, Octave is started with the shell command
octave. Octave displays an initial message and then a prompt
indicating it is ready to accept input. You can begin typing Octave
commands immediately afterward.

If you get into trouble, you can usually interrupt Octave by typing
`Control-C` (written `C-c` for short). `C-c` gets
its name from the fact that you type it by holding down <CTRL> and
then pressing <c>. Doing this will normally return you to Octave's
prompt.

To exit Octave, type `quit`, or `exit` at the Octave prompt.

On systems that support job control, you can suspend Octave by sending
it a `SIGTSTP` signal, usually by typing `C-z`.
